# A Descriptive Study of Caregivers’ Awareness, Practices, Challenges Faced, and Methods of Adherence to the Ketogenic Diet in Children with Refractory Epilepsy

**DOI:** 10.3390/nu17162605

**Published:** 2025-08-11

**Authors:** Nora Alafif, Fahdah AlShaikh, Monirah Albloushi, Nawaf W. Alruwaili, Waad Alfawaz, Fay Almutairi, Entisar Alhany, Jamilah Ghazwani, Nesreen Alduhaim, Shabana Tharkar, Khalid M. Almutairi

**Affiliations:** 1 Department of Community Health Sciences, College of Applied Medical Sciences, King Saud University, Riyadh P.O. Box 11433, Saudi Arabia; nalafeef@ksu.edu.sa (N.A.); nalruwaili@ksu.edu.sa (N.W.A.); walfawaz@ksu.edu.sa (W.A.); jo46.ya@gmail.com (J.G.); ngot777@gmail.com (N.A.); kalmutairim@ksu.edu.sa (K.M.A.); 2 College of Nursing, King Saud University, Riyadh P.O. Box 11433, Saudi Arabia; malbloushi@ksu.edu.sa; 3 Clinical Nutrition Department, King Fahad Specialist Hospital, Dammam P.O. Box 32253, Saudi Arabia; fjalmutairi2@moh.gov.sa (F.A.); ealhany@moh.gov.sa (E.A.)

**Keywords:** caregivers, awareness, practices, ketogenic diet, children, refractory epilepsy

## Abstract

**Background**: Refractory epilepsy refers to a type of epilepsy in which anti-epileptic medications do not yield results, necessitating alternative medical interventions. The ketogenic diet (KD) is utilized as a non-pharmacologic treatment for refractory epilepsy. This study aims to assess details regarding caregivers’ awareness and practices, challenges faced, and other details regarding the KD as a treatment option for children with refractory epilepsy. **Methods**: A cross-sectional study included 15 caregivers of children with refractory epilepsy who adhere to the KD. Data were collected using an online, self-administered questionnaire including socio-demographic characteristics and descriptive aspects of KD on 15 caregivers. **Results**: Caregivers’ awareness scores varied, over half of the caregivers (53.3%) demonstrated low awareness levels, scoring below the midpoint on the awareness scale, while both caregivers’ adherence (t value = 31.5; *p* < 0.001) and patients’ adherence levels (t value = 26.1; *p* < 0.001) significantly exceeded the minimum expected thresholds, indicating effective implementation of the diet protocols. Challenges faced by caregivers were categorized as follow: logistical challenges (e.g., issues related to KD availability, cost, and weighing (15.6%)), behavioral challenges (e.g., avoiding prohibited foods (18.8%), feelings of hunger, the social environment, and never eating without caregivers), and medical challenges (e.g., frequent blood glucose monitoring (25%)). The most common side effects of the KD were gastrointestinal symptoms, including constipation (33.3%) and gastroesophageal reflux disease (GERD). Physical symptoms such as dyslipidemia were also reported by 13.3% of participants. In terms of neurological and behavioral improvements, the three most reduced symptoms following KD adoption were hyperactive attention deficit (25%), tremors (20%), and insomnia (20%). **Conclusions**: This study reveals a significant gap between high adherence and low awareness of the KD among caregivers of children with refractory epilepsy. While adherence is crucial for the success of KD, this discrepancy highlights the need for comprehensive caregiver education that not only focuses on practical implementation but also on the underlying rationale of the diet. This study underscores the importance of multidisciplinary support, by including dietitians, to bridge the knowledge gap. These findings suggest that dietary education programs should aim to improve caregivers’ understanding, ultimately enhancing adherence and long-term outcomes. Future research should explore the psychosocial factors influencing adherence and the role of trust in healthcare professionals in shaping caregiver behaviors. The findings also call for further exploration into the impact of caregiver education on child health outcomes in the long term. The varied knowledge among caregivers indicates a need for further educational interventions or increased visits from dietitians. Strategies need to be framed to overcome the challenges faced and improve adherence.

## 1. Introduction

Epilepsy is a serious neurological disorder that affects nearly 50 million people of all ages worldwide [1]. It ranks as the fourth most common brain disorder [2] and is characterized by recurrent seizures, which can appear as behavioral or motor disturbances [3]. In Saudi Arabia, it affects approximately 6.54 per 1000 adults and children [4]. Refractory epilepsy (RE) is a type of epilepsy where seizures persist despite the use of various anti-seizure medications [4]. The KD is an established and effective therapy for drug-resistant epilepsy, working by inducing ketosis to reduce seizure frequency and severity. However, its implementation poses challenges, particularly related to inadequate hydration and fiber intake, which can lead to gastrointestinal issues like constipation and affect adherence. Effective management of these factors, along with targeted education for caregivers and healthcare professionals, is essential to support the diet’s long-term success [5]. Despite many available anti-seizure drugs, a significant number of epilepsy patients continue to have seizures and do not respond to current treatments. A recent meta-analysis estimated the incidence of RE at 25.0% in studies involving children, compared to 14.6% in adult or mixed-age studies [6]. The KD has emerged as a potential treatment option for these epilepsy cases [7]. Recent studies have highlighted the metabolic mechanisms of the KD, supporting its effectiveness in reducing seizure frequency and improving quality of life for children with RE. These findings reinforce the necessity of careful monitoring and education in its application [8].

The KD has received significant attention for its ability to potentially decrease the frequency and severity of seizures in specific individuals with RE [9]. However, following this diet may be challenging because of its strict rules and the requirement of closely tracking macronutrient consumption. The diet could also lead to different side effects, such as issues with the digestive system, abnormal levels of lipids in the blood, and a lack of essential nutrients [10]. Hence, it is important to consult with diverse healthcare professionals, such as neurologists, dietitians, and other specialists, when considering starting the KD to ensure it is implemented, monitored, and managed effectively to prevent any possible adverse outcomes [11]. The goal was to examine the effectiveness of the KD on children with different types of drug-resistant epilepsy. They found that the KD therapy was effective in about two-thirds of children with drug-resistant epilepsy [12].

Given this evidence, wide-ranging considerations are necessary to effectively screen the caregivers’ use of the KD in managing the frequency and severity of seizures in children with RE. The current literature lacks an understanding of the caregivers’ awareness and practices toward using the KD in treating children with RE within the Saudi Arabian context. This is the first study to measure the extent of the caregivers’ awareness, the extent of adherence, and the difficulties in implementing the KD, as well as methods used, and the effect of KD as a treatment for children with RE. By addressing these knowledge gaps, the study seeks to improve the benefits of using the KD as a treatment and open new opportunities for research and awareness programs for caregivers of children with RE.

## 2. Materials and Methods

### 2.1. Study Design and Sample Size

This analytical, cross-sectional study assesses the caregivers’ awareness and attitude toward the use of the KD on children with RE. The study was conducted from August 2023 to May 2024, and the participants were recruited in collaboration with the Saudi Epilepsy Society. The study utilized a purposive sampling strategy, targeting caregivers of children diagnosed with RE who were actively following the KD as advised by healthcare professionals. Recruitment was conducted in collaboration with the Saudi Epilepsy Society, who distributed the electronic survey to caregivers meeting these criteria. The caregivers who were included in the study were the only caregivers that fit the following inclusion criteria: caregivers of children aged 0–18 years diagnosed with RE by a healthcare provider; children actively following the KD at the time of the study; and caregivers who had the ability to comprehend and complete the survey in Arabic. All those caregivers of RE who did not use any treatment or did not follow a KD were excluded from the study. The collected data included the attitude and awareness of the caregivers of RE who were using the KD [13]. The electronic survey was given to the Saudi Epilepsy Society and then distributed to caregivers of RE [14]. The sample size was calculated by looking at a sample size of five similar studies and the scientific literature [15,16], where the sample size was found to range from 17 to 36 subjects, and a sample size of 25% (29 participants) was estimated to reach informational power. The total sample size included in this study was thirty-two caregivers of RE, but only 15 met the inclusion criteria as those who followed the KD as a treatment. Informed consent was obtained after explaining all the steps of the research on the first page of the questionnaire. An e-signature was taken before participating in the study.

### 2.2. Assessment Tools

The self-administered online questionnaire was adapted from a previously validated tool and reviewed for face validity by experts in clinical nutrition and nursing. A formal pilot study was not conducted due to difficulty accessing the specific caregiver population [17]. The data collection tool was an online, self-administered questionnaire with four main sections designed based on a validated survey from a previous study. The first section included the socio-economic and demographic characteristics of the population; the second section included the descriptive aspects of the KD; the third section included the KD practices and approaches of the caregivers; and the last section included the KD knowledge of the caregivers and their adherence to KD, for both the patients and caregivers [18].

The first section of the questionnaire includes a question about categorizing outcomes related to the KD and the timing of data collection. Each category was defined as follows: the 1–2 seizure per week category refers to caregivers’ reporting that their children experience 1 to 2 seizure episodes weekly, in contrast to their baseline frequency. The 3–4 seizures per week category indicates that caregivers are reporting that their children are experiencing 3 to 4 seizure episodes weekly. The shortened seizure duration category pertains to caregivers who reported a noticeable reduction in the length of individual seizure episodes [19].

To capture significant improvements in seizure characteristics—such as reductions in seizure duration or intensity as reported by caregivers—the current study included a questionnaire item on the “effectiveness of KD,” defined as a >50% reduction in seizures and a reduction or cessation of antiseizure medications (ASMs) within three months of initiating the diet. The questionnaire utilized a previously validated instrument that has been applied in similar studies to evaluate caregivers’ knowledge and adherence to therapeutic diets. Both awareness and adherence were assessed on a 0–10 scale. An awareness score above 5 indicated high awareness, while a score below 5 denoted low awareness. Adherence was also measured on the same scale, with higher scores signifying greater compliance with the KD. Minor modifications were made to ensure relevance and clarity, and the revised questionnaire was reviewed for face validity by experts in clinical nutrition and nursing [20]. However, due to the limited sample size and the difficulty accessing this specific caregiver population, we were unable to conduct a pilot study with the same group of caregivers.

A question was included in the survey to assess the sources of information caregivers used to learn about the KD. Caregivers were asked to identify whether they learned about the diet through healthcare professionals, the internet, social media, or other sources, providing insight into the factors influencing their knowledge and adherence.

### 2.3. Statistical Analyses

The collected data were analyzed using SPSS software (version 28). Knowledge and adherence questions were rated 0 to 10. A positive score of five or greater was categorized as high. Descriptive statistics were used to measure the demographic characteristics of the studied population. A one-sample *t*-test was used to find the correlation between the awareness and adherence of caregivers with RE children who were following the KD. Although the sample size was small the use of *t*-tests was chosen to assess the correlation between caregivers’ knowledge and adherence, based on previous studies with similar sample sizes. However, we acknowledge that larger samples would provide greater statistical power and more robust conclusions. A *p*-value of ≤0.001 was considered significant. Given the small sample size (n = 15), we acknowledge that the assumptions for *t*-tests, including normality, may not be fully met. However, we chose to use *t*-tests as the data followed a general trend of normal distribution in preliminary visual assessments (e.g., histograms, Q-Q plots). In the future, we recommend using non-parametric tests (e.g., the Mann–Whitney U test) for smaller samples to avoid assumption violations. Further statistical techniques could be explored with larger samples

A one-sample *t*-test was used to assess the correlation between caregivers’ knowledge and adherence to the KD. Given the small sample size (n = 15), the high *t*-values (31.5 and 26.1) reflect the large differences between the groups’ means and the small variability within the sample. However, these results should be interpreted cautiously due to the sample’s limited size, which may influence the statistical power and generalizability of the findings.

Future studies should consider including a general assessment of physical activity levels in both caregivers and children, as physical activity may influence both the effectiveness of the KD and overall well-being during diet therapy. Including this data would provide a more comprehensive understanding of the factors affecting diet adherence and health outcomes.

## 3. Results

### 3.1. Demographics

In the initial phase of data collection, responses from 32 participants were evaluated. Of the 32 respondents, only 15 were implementing the KD with their children. Over half of the caregivers (53.3%) demonstrated low awareness, scoring below the midpoint on the awareness scale. The demographic and clinical characteristics of the children of caregivers following the KD for RE are presented in Table 1. Gender distribution is nearly balanced, with slightly more males (n = 8, 53.3%). The socio-economic status of most caregivers is middle income (n = 13, 86.7%), and no family history of epilepsy (73.3%).

### 3.2. Side Effects of the KD

Six (32%) of the fifteen caregivers reported experiencing side effects while adhering to the KD, whereas the remaining nine (68%) caregivers did not report any side effects. The most common side effects noted by the caregivers were gastrointestinal symptoms, including constipation (33.3%) and GERD. Physical symptoms such as dyslipidemia were also reported by 13.3% of the participants. In terms of neurological and behavioral improvements, the three most reduced symptoms following KD adoption were hyperactive attention deficit (25%), tremors (20%), and insomnia (20%). The challenges faced by caregivers were categorized into three main groups:

- Logistical challenges: Issues related to the availability of ketogenic products, cost, and the need to weigh food portions (15.6%).
- Behavioral challenges: Difficulties in managing forbidden food intake and feelings of hunger (18.8%).
- Medical challenges: Frequent blood glucose monitoring (25%).
- The symptoms observed following the adoption of the KD can also be categorized as follows:
- Gastrointestinal symptoms: Constipation (33.3%) and GERD.
- Neurological/baehaviorl symptoms: Hyperactive attention deficit (25%), tremors (20%), and insomnia (20%).
- Physical symptoms: Dyslipidemia (13.3%).

### 3.3. Knowledge About the KD

All participants confirmed that they received education and information regarding the KD, with instructions provided by a dietitian, which represented the highest percentage at 100%. Additionally, four out of fifteen caregivers stated that they received education from a physician, in contrast to those who received it from a nurse or a multidisciplinary team. Eight out of fifteen caregivers reported that the duration of the education they received was less than 60 min, accounting for 53.5% of the total participants. In comparison, two (13.5%) indicated that the duration of education was between 60 and 120 min, and another two (13.5%) reported 121–180 min, which represents the lowest percentage compared to the three individuals who received extensive education lasting more than 180 min.

Table 2 shows the knowledge and adherence scores, along with the correlation between them. Eight caregivers scored less than five in Group I. In contrast, caregiver adherence was higher (13 vs. 2), as was patient adherence (12 vs. 3), indicating effective diet implementation despite differences in knowledge.

Adherence levels for caregivers (mean difference 2.87, t = 31.553, 95% CI: 2.67–3.06) and patients (mean difference 2.8, t = 26.192, 95% CI: 2.57–3.03) were similarly high.

### 3.4. Challenges Encountered

Table 3 highlights the medical, behavioral, and logistical challenges of managing the KD for children with RE. Among the medical challenges, frequent blood glucose monitoring was reported by 8 caregivers (25%) and emerged as the most significant concern. Behavioral challenges, such as difficulties in managing forbidden food intake, were also commonly reported. In addition, several logistical challenges, including the cost of ketogenic products, their limited availability, and the need to weigh food portions, affected more than one-third of the participants. These findings underscore the multifaceted difficulties involved in daily KD adherence and highlight the need for additional caregiver support, structured education, and practical resources.

### 3.5. Methods of KD Adherence

**Figure 1:**
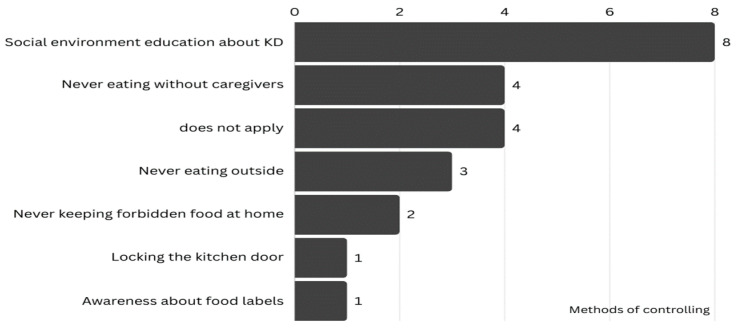
Methods of adhering to the KD.

Figure 1 illustrates methods used by caregivers to control forbidden food intake. The most common method to control the children’s intake of prohibited foods was through social environment education, followed by never eating without caregivers. They have knowledge and awareness of nutrition labels, which may help control food intake in their children with RE. Overall, four caregivers did not apply any method of adherence.

Several strategies have been reported for addressing food refusal in children on the KD. Half the participants found encouragement and retrying effective, while others mixed the food with favorites (five participants) or froze/served it as a drink (three participants).

### 3.6. Reduction of Symptoms Following KD

The reduction in various symptoms following the adoption of the KD among children with RE is shown in Table 4.

Hyperactive attention deficit and insomnia, each reported at 25% and 20%, were the two most frequently reduced neurologic symptoms following KD. When considering multiple symptoms per child, these symptoms correspond to 33.3% and 26.7% of all instances. Overall, the table highlights KD’s potential role in alleviating diverse epilepsy-related symptoms beyond seizure control, reflecting its multifaceted neurological benefits.

## 4. Discussion

The study examined caregivers’ practices, adherence, and the effects of the KD for children with RE. Results suggest variability in the level of knowledge among caregivers, highlighting a potential area for targeted educational efforts. Notably, all caregivers reported that they gained their knowledge of this nutritional strategy from a nutritionist or other medical professional. However, in contrast to their knowledge, caregiver adherence to the diet was higher. This strong adherence is promising, as it indicates most caregivers effectively follow the diet’s protocols. Similarly, patient adherence was also categorized as high. This high level of adherence among patients is crucial for the success of the KD in managing epilepsy. The contrast between the relatively limited distribution of caregivers’ knowledge and the high level of adherence suggests that even when caregivers do not feel fully informed, they may still comply effectively with dietary requirements. This outcome may be attributed to external support systems, practical training, or strong motivation from both caregivers and patients. Despite variation in self-reported knowledge, identifying the factors contributing to high adherence can inform the development of more effective educational and support programs. As previously reported, children undergoing ketogenic dietary therapy may experience adverse effects, highlighting the importance of regular monitoring and individualized dietary adjustments to ensure both safety and tolerance [1]. Constipation emerged as the most frequently reported side effect among caregivers in the current study, consistent with findings from earlier ketogenic diet research. This symptom is likely exacerbated by insufficient fluid intake and low dietary fiber consumption, both of which are commonly associated with ketogenic dietary protocols. During ketosis, increased fluid excretion occurs due to reduce glycogen storage and insulin levels, leading to a higher risk of dehydration if fluids are not sufficiently replenished [1]. Dehydration contributes to hard stool consistency and reduces intestinal motility, making defecation more difficult [2]. Simultaneously, the KD restricts high-fiber carbohydrate-rich foods such as fruits, legumes, and whole grains, resulting in a lower fiber intake [3]. Fiber is essential for increasing stool bulk and promoting peristalsis; when combined with poor hydration, the risk of constipation increases significantly [1,3]. These observations underscore the importance of regular dietitian-led counseling to ensure caregivers are informed about incorporating KD-compliant fiber sources and maintaining adequate hydration as part of a comprehensive management strategy. A major limitation of this study is the small sample size (n = 15), which may limit the statistical power and generalizability of the findings. Future studies with larger sample sizes are recommended to better assess the impact of the KD on caregivers and patients.

An interesting finding of this study is the discrepancy between caregivers’ high adherence to the KD protocols and their low awareness or understanding of the diet’s theoretical aspects. This discrepancy suggests that adherence may be influenced by factors other than knowledge alone. One possible explanation is that caregivers may strictly follow the protocols based on trust in healthcare professionals or instructions from dietitians, without necessarily understanding the underlying principles of the diet. This phenomenon has also been noted in previous studies where caregivers prioritize following medical advice over understanding the scientific basis of the diet [17]. Another factor contributing to high adherence may be the motivation to improve the child’s health outcomes, which could drive caregivers to adhere to the diet’s demands even without fully grasping its mechanisms. Future interventions should not only focus on ensuring adherence to the diet but also aim to educate caregivers about the rationale behind the KD to improve their understanding and engagement.

Some studies reported that children may occasionally experience challenges with hunger that affect their adherence to the diet [18]. To maintain control over their child’s diet, some caregivers focused on educating the child’s community about the diet, while others emphasized close supervision and strict food restrictions.

Regarding the effectiveness of the KD, positive results were observed after KD adherence. Some children experienced a decrease in epilepsy seizures, and in certain cases, seizures completely ceased. Additionally, the diet showed potential to alleviate various symptoms related to epilepsy, like insomnia and hypersalivation which showed moderate reductions, suggesting that the KD is effective in reducing these symptoms. Other symptoms, such as tremors, hyperactive attention deficit, and eye twitching, exhibit weak negative correlations, indicating a minimal impact on the duration of these symptoms. These results suggest a diverse effect of the treatment method on the efficacy of duration across different symptoms, highlighting the complexity of treatment outcomes. In a comparable study involving 126 patients, limited awareness of the KD was found, with only one-third of participants being familiar with it. Among them, nine patients used the diet specifically to manage epilepsy, resulting in reduced seizures and improved sleep quality. Further research with larger samples and more detailed symptom tracking would be valuable in clarifying these relationships.

In one study assessing caregiver perceptions of dietary therapies for children with epilepsy, most caregivers expressed satisfaction, reporting strong support during treatment, improved quality of life, and overall contentment with the diet [19]. Another study found that parents of children with epilepsy generally had a positive perception of the KD, though many expressed concerns about its potential long-term effects [20].

The challenges faced with the KD merit special acknowledgment. In addition to glucose monitoring, limited availability, and the high cost of KD products were some of the difficulties caregivers encountered. The findings are consistent with previous studies that reported similar challenges associated with the ketogenic diet [17]. Moreover, the results suggest that the social environment and interpersonal interactions play a pivotal role in supporting adherence, with eating under caregiver supervision identified as a key strategy. In contrast, other studies have emphasized food label awareness, avoiding meals outside the home, and strict guidance by caregivers as the most effective methods for preventing the consumption of non-compliant foods.

The present study has some limitations. The sample size was not sufficient. It was difficult to reach the targeted group, so participants were recruited through the Saudi Epilepsy Society by contacting all registered families. The results cannot be generalized to a larger population because most caregivers were mothers with a high level of education.

## 5. Conclusions

These results emphasize the significance of providing practical support to caregivers while also revealing a gap between knowledge and behavior that merits further investigation. Exploring the reasons behind caregivers’ high adherence rates despite limited awareness could shed light on factors such as trust in healthcare professionals, levels of health literacy, and culturally informed decision-making. Addressing these aspects may facilitate the development of more targeted and culturally appropriate interventions aimed at narrowing the divide between knowledge and practice. This study highlights that while caregivers demonstrated high adherence to KD protocols for children with RE, their overall awareness and understanding of the diet were variable. This discrepancy suggests a need for targeted strategies to both sustain adherence and enhance caregivers’ knowledge. Based on the findings, several targeted strategies are proposed to enhance caregiver support and optimize the implementation of the ketogenic diet. First, structured educational programs should be developed to strengthen caregivers’ theoretical and practical understanding of the ketogenic diet. These programs should emphasize the rationale behind dietary protocols, long-term management considerations, and the identification and management of potential side effects. Second, regular multidisciplinary follow-up appointments involving dietitians, neurologists, and nursing professionals are recommended to provide continuous education, monitor adherence, and address implementation challenges. These regular follow-up sessions can be conducted once every 4 to 6 weeks to assess adherence, address concerns, and reinforce dietary understanding. Depending on the family’s geographic location and accessibility, these sessions can be offered in person or through telehealth platforms, which may improve caregiver engagement and continuity of care, especially in underserved or remote areas. Development of accessible resources: Culturally and linguistically tailored resources, including printed guides, videos, and mobile applications, should be made available to help caregivers manage the diet effectively at home. Addressing logistical and financial barriers: Healthcare systems should work towards improving the availability and affordability of ketogenic products and monitoring tools, recognizing the financial and logistical burdens reported by caregivers. Facilitating peer support: Establishing caregiver support groups or online forums may offer valuable platforms for sharing practical advice, emotional support, and successful strategies for overcoming challenges associated with KD adherence. This study did not assess caregivers’ or patients’ levels of physical activity, which may influence outcomes such as adherence, gastrointestinal symptoms, and overall well-being during KD therapy. Future studies should consider including physical activity assessments as part of a broader lifestyle evaluation. Additionally, while recording the healthcare professionals who provided dietary education, we did not capture data on caregivers’ initial sources of information about the KD (e.g., internet, social media, and peer networks). Understanding these information pathways may offer valuable insights into caregivers’ decision-making and trust in medical guidance and should be explored in future research. These recommendations aim to strengthen caregiver capacity, promote sustained adherence, and ultimately improve health outcomes for children with RE. Future research should explore the long-term effects of these strategies and consider qualitative approaches to further understand caregivers’ experiences and needs. Based on the study’s findings, several strategies are recommended to enhance caregiver adherence to the KD. These include the development of structured and culturally appropriate educational programs that explain both the practical and theoretical aspects of the diet, along with regular multidisciplinary follow-ups involving dietitians and neurologists to support ongoing adherence. Additionally, accessible resources such as mobile applications, instructional videos, and printed guides should be provided to assist caregivers at home. Efforts should also be made to reduce financial and logistical barriers through improved healthcare system support, and peer-led support groups, either online or in local communities, should be established to foster shared learning and emotional encouragement.

Dietitians should be involved in regular follow-up sessions to monitor adherence and provide guidance. These sessions should occur every 4 to 6 weeks, with a combination of in-person visits for hands-on support and online consultations to provide flexibility and accessibility, especially for caregivers in remote areas. The frequency of these visits should be tailored to each caregiver’s needs and the child’s condition. Regular consultations will help reinforce caregivers’ understanding of the KD and provide the necessary support to address any challenges they encounter in managing the diet at home.

In addition to providing caregivers with proper education and support, it is crucial to develop structured, scientifically accurate counseling programs for healthcare professionals. These programs will ensure that medical practitioners have reliable, evidence-based information, countering the growing influence of misleading online sources and equipping them to offer more effective guidance to families managing the KD for epilepsy.

## Figures and Tables

**Table 1:** Demographics of the participants (n = 15).

Variable	Category	Number	Percent
Gender	Female	7	46.7
	Male	8	53.3
Socio-Economic Status	Low	2	13.3
	Middle	13	86.7
	High	0	0
Family History of Epilepsy	Parent or Siblings	2	13.3
	Other Relatives	2	13.3
	No	11	73.3
Educational Status of Mother	Less Than High School	1	6.7
	High School Graduate	2	13.3
	4 Year College Degree	12	80
Working Status of Mother	Working	4	26.7
	Not Working	11	73.3
The Effect of Following the KD	1–2 Seizures Per Week	5	33.3
	3–4 Seizures Per Week	2	13.3
	Shortened Seizure Duration	5	33.3
Duration Between Effectiveness and Beginning of KD	1 Month	10	66.7
	2 Months	3	20.0
	3 Months	1	6.7
	6 Months	0	0
	9 Months	1	6.7

**Table 2:** Knowledge scores and one-sample *t*-test showing correlation between awareness and adherence.

Variables	Knowledge Score Group I ≤ 5 n = 15	Knowledge Score Group II > 5 n = 15
Caregivers’ knowledge	8 (53.3)	7 (46.7)
Caregivers’ adherence	2 (13.3)	13 (86.7)
Patient adherence	3 (20)	12 (80)
Correlation between knowledge and adherence		
Variables	t value; *p* value	Confidence interval
Caregivers’ knowledge and caregivers’ adherence	31.55; 0.000	2.67–3.04
Caregivers’ knowledge and patient adherence	26.19; 0.000	2.57–3.02

**Table 3:** An overview of the various challenges encountered by caregivers while managing the KD for children with RE (n = 15).

Difficulties of KD	Number	Percent
Availability of Ketogenic Products	5	15.6
Frequent Blood Glucose Monitoring	8	25
Feeling of Hunger	3	9.4
Weighing Food	5	15.6
Forbidden Food Management	6	18.8
Product Cost	5	15.6
Total	32	100

**Table 4:** Various symptoms following the adoption of the KD among children with RE (n = 15).

Decrease Symptoms After KD	Number	Percent
Tremor	4	20
Hyperactive Attention Deficit	5	25
Eye Twitching	2	10
Hypersalivation	1	5
Anorexia	3	15
Insomnia	4	20
Aggressive Behavior/Anger Outbursts	1	5

## Data Availability

The datasets generated during and/or analyzed during the current study are available from the corresponding author (Correspondence: stharkar@ksu.edu.sa; Tel.: +966-537998800) on reasonable request due to privacy reasons.
